# Inherited Thyroid Tumors With Oncocytic Change

**DOI:** 10.3389/fendo.2021.691979

**Published:** 2021-06-09

**Authors:** Marcelo Correia, Ana Rita Lima, Rui Batista, Valdemar Máximo, Manuel Sobrinho-Simões

**Affiliations:** ^1^ Cancer Signalling and Metabolism, Instituto de Investigação e Inovação em Saúde (i3S), Universidade do Porto, Porto, Portugal; ^2^ Cancer Signalling and Metabolism, Institute of Molecular Pathology and Immunology of the University of Porto (IPATIMUP), Porto, Portugal; ^3^ Faculty of Medicine of the University of Porto (FMUP), Porto, Portugal; ^4^ Department of Pathology, Faculty of Medicine of the University of Porto (FMUP), Porto, Portugal; ^5^ Department of Pathology, Centro Hospitalar e Universitário São João (CHUSJ), Porto, Portugal

**Keywords:** oncocytic thyroid tumors, Hürthle cell, TCO locus, mitochondria, genetic predisposition

## Abstract

Familial non-medullary thyroid carcinoma (FNMTC) corresponds to 5-10% of all follicular cell-derived carcinoma (FCDTC). Oncocytic thyroid tumors have an increased incidence in the familial context in comparison with sporadic FCDTC, encompassing benign and malignant tumors in the same family presenting with some extent of cell oxyphilia. This has triggered the interest of our and other groups to clarify the oncocytic change, looking for genetic markers that could explain the emergence of this phenotype in thyroid benign and malignant lesions, focusing on familial aggregation. Despite some advances regarding the identification of the gene associated with retinoic and interferon-induced mortality 19 (GRIM-19), as one of the key candidate genes affected in the “Tumor with Cell Oxyphilia” (TCO) locus, most of the mutations follow a pattern of “private mutations”, almost exclusive to one family. Moreover, no causative genetic alterations were identified so far in most families. The incomplete penetrance of the disease, the diverse benign and malignant phenotypes in the affected familial members and the variable syndromic associations create an additional layer of complexity for studying the genetic alterations in oncocytic tumors. In the present review, we summarized the available evidence supporting genomic-based mechanisms for the oncocytic change, particularly in the context of FNMTC. We have also addressed the challenges and gaps in the aforementioned mechanisms, as well as molecular clues that can explain, at least partially, the phenotype of oncocytic tumors and the respective clinico-pathological behavior. Finally, we pointed to areas of further investigation in the field of oncocytic (F)NMTC with translational potential in terms of therapy.

## Introduction

Thyroid tumors with oncocytic features are referred in the literature as oncocytic, Hürthle cell, oxyphilic, eosinophilic, and even mitochondrion-rich thyroid tumors. The oncocytic tumors show a peculiar granular eosinophilic staining and swollen cells, which result from the huge accumulation of abnormal and dysfunctional mitochondria in the cells’ cytoplasm. Since the 2017 WHO classification, Hürthle cell tumors (HCT), adenomas and carcinomas with follicular characteristics (HCA and HCC, respectively) are a separate diagnostic category, while the oncocytic variants of papillary, medullary and poorly differentiated carcinoma were kept as a subset of these diagnostic categories (1). In the present review we have kept, whenever possible, the utilization of oncocytic tumors as a common denomination.

Non-medullary thyroid carcinoma (NMTC) is the most common group of thyroid cancers (TC), accounting for more than 90% of the cases, and it is the main responsible for the increase in TC incidence (2, 3). The evidence of a familial aggregation of NMTCs is accumulating over the years with a prevalence of 5-10% in different series on record (4). Epidemiological studies showed an eight to ten times higher relative risk for the development of the disease in first-degree relatives of TC patients (5–11).

## Non-Syndromic Genetic Predisposition to Oncocytic Change

In a non-syndromic context, named as familial non-medullary thyroid carcinoma (FNMTC), oncocytic phenotype is usually present in at least one individual in the great majority of families. Oncocytic tumors are also present in a syndromic context of familial TC coexisting with other subtypes of carcinomas in different organs (e.g., Hereditary Paraganglioma-Pheochromocytoma, Cowden syndrome, Birt-Hogg-Dubé syndrome, Von Hippel Lindau syndrome and Hyperparathyroidism- Jaw syndrome) (12). Familial aggregation has been found in several families, regardless of oncocytic tumor presence, but the genetic alterations underlying FNMTC are still poorly understood (5). Familial aggregation of thyroid tumors, oncocytic and non-oncocytic, seems to follow a genetic and phenotypic heterogeneous pattern that challenges the identification of the genetic causative event and the establishment of a phenotype-genotype correlation. The multiplicity of genetic and environmental factors that contributes to the onset of familial oncocytic tumors presents as a major challenge for the scientific community. Several families have been described in the literature, but very little has evolved towards the identification of a common denominator genetic event that would allow the identification of the hereditary causality. Likewise, there is scarce evidence about the oncocytic onset etiopathogenesis. Genome Wide Association Studies (GWAS) have been performed in probands and relatives of FNMTC families in an effort to establish the causative risk transmission alleles. Population common single nucleotide polymorphisms (SNP) were associated with FNMTC, but the weak association demonstrated to date has prevented the identification of a main genetic event (driver alteration) so far. Common SNPs fail to answer a crucial question: How come the disease is not developed in cases of individuals without thyroid disease in whom the common SNP variant is also present (6, 8, 9, 13). So far, the most robust described genomic area associated with a familial aggregation of oncocytic tumors was described by Canzian et al. (14). The authors successfully mapped genetic events that increase the susceptibility for oncocytic thyroid tumors in the “Tumor with Cell Oxyphilia” (TCO) locus at 19p13.2, by microsatellite linkage analysis in a French family with high occurrence of oncocytic tumors (14). Later, McKay et al. validated the association of TCO locus with increased susceptibility to the oncocytic phenotype in 10 additional families, nine of them with one or more individuals presenting oncocytic tumors (15).

The study of TCO established the basis for further genetic associations. Some authors attempted, and are attempting, to pinpoint the causative genes at TCO locus that may explain oncocytic tumor aggregation and its onset on families (**Table 1**).

**Table 1:** Summary of genetic linkage and gene mutations that are associated with hereditary thyroid tumors with oncocytic change.

Thyroid tumor histotype	Inheritance	Locus	Candidate gene	Gene mutation	Refs
Individuals from one family affected with multinodular goiter and papillary thyroid carcinoma, with and without cell oxyphilia	Germline (autosomal dominant)	TCO	Linkage to a gene on 19p13.2 – TCO locus		(14)
Oncocytic thyroid carcinomas (follicular and papillary)	Germline and somatic	TCO	GRIM-19	- G264C; K88N (germline)	(16)
				- C77T; A26V (somatic)	
				- A247G; S83G (somatic)	
				- G593C; R198P (somatic)	
Individuals from families affected with thyroid tumors with and without cell oxyphilia	Germline and somatic	TCO	TIMM44	- C925A; P308Q (co-segregation with TCO)	(17)
				- G1274A; silent (co-segregation with TCO)	
				- G344A; silent (somatic)	
				- C1307T; silent (somatic)	
Two oncocytic thyroid carcinomas and one oncocytic thyroid adenoma	Germline	TCO	MYO1F	- G400A; G134S	(18)

### GRIM-19

The gene associated with retinoic and interferon-induced mortality 19 (GRIM-19, or also named NADH:ubiquinone oxidoreductase subunit A13, NDUFA13), located in the TCO locus at 19p13.2, is currently known an association between the oncocytic phenotype and a specific gene in TCO locus. In 2005, Maximo et al. identified a germline mutation in GRIM-19 in an individual who presented oncocytic variant of papillary thyroid carcinoma (OV-PTC) on a background of multiple oncocytic cell nodules (16), but the authors did not find loss of heterozygosity for GRIM-19 in tumor tissues. GRIM-19 was as an excellent candidate as there is enough evidence linking the onset of cell death induced by gamma interferon and retinoic acid and presents crucial mitochondrial functions that serve as a scaffold protein in the mitochondrial respiratory complex I (19). Supporting the alterations(s) of GRIM-19 gene in the tumorigenic process, the encoded protein was found downregulated in several human tumors (20–23). *In-vitro* studies have shown that GRIM-19 downregulation increased proliferation and cell metabolic shift to a more glycolytic state (24, 25), typical of tumor cells. In an elegant *in-vivo* study, abrogation of a single allele of GRIM-19 in C57BL6 mice increased the susceptibility towards tumorigenesis (26).

Some studies have reported that the disruption of GRIM-19 function may have a direct relation with abnormal mitochondrial function and morphology (19, 26) that can contribute to explain the oncocytic phenotype in TC patients. Also, GRIM-19 missense somatic mutations were detected in oncocytic tumors, being absent in non-oncocytic tumors (16).

### TIMM44 and MYO1F

Other attempts to associate the TCO locus with a specific gene have identified both Translocase of Inner Mitochondrial Membrane 44 (TIMM44) and Myosin-IF (MYO1F) (17, 18). Similar to GRIM-19, TIMM44 is located in the TCO region (19p13.2). A study by Bonora et al. associated the presence and co-segregation of two specific SNPs (through linkage association) in TIMM44 to an increased predisposition to oncocytic tumors (17). This was an important finding, but as in GRIM-19 alterations, it failed to detect the presence of TIMM44 specific variants and/or new variants in other families, rendering the importance of TIMM44 gene for genetic susceptibility to oncocytic phenotype uncertain. The importance of *in-vitro* and *in-vivo* validation is well shown in the effort to better elucidate the aforementioned finding (17).

Diquigiovanni et al. associated the presence of a novel alteration detected in MYO1F gene to predisposition for oncocytic tumors in one affected family (18). The authors performed *in-vitro* studies to ascertain and confirm the relevance of MYO1F in the tumorigenic process. FRTL-5 thyroid rat cell line was modified to harbor the MYO1F G400A mutation, revealing an altered mitochondrial morphology and mass, strongly suggesting that MYO1F may play a role in the establishment of the oncocytic phenotype (18).

Other possible genes mapped to the TCO locus have also been screened for the presence of alterations and, most importantly, for the segregation of oncocytic tumors. Some mutations were reported (genes ELAVL1, CCL25, ADAMTS10, ANGPTL4, LASS1, LASS4, RAB11B, MARCH2, EDG5, RAB1, MUC16), but they did not seem to predispose for familial tumors with oncocytic change (17, 27).

## Syndromic Associations With Genetic Predisposition

Several associations with an increased risk of development of oncocytic tumors were found outside the TCO locus spectrum. Common examples rely on the association between the oncocytic phenotype and co-occurrence with other cancer phenotypes in the same families in a hereditary syndromic context. An example is the description of a patient with Cowden syndrome previously presenting a PTEN germline mutation, who harbored an oncocytic tumor presenting a somatic alteration in the Folliculin (FLCN) gene (28). In this tumor, the authors described this “double heterozygosity” as the only detectable tumorigenic hit in a context of high chromosomal stability. This interesting finding strongly suggested a role of PTEN/FLCN synergistic effect in syndromic oncocytic tumorigenesis (28).

Although the aforementioned syndromic finding presents exciting and intriguing alternatives explaining the predisposition to oncocytic tumors, most of the mutations identified follow a pattern of “private mutations”, being exclusive to a specific family, or present in only a very small number of families. In most of the families with oncocytic tumors, causative genetic alterations have not been identified to date (4, 11, 14). Genetic alterations associated with oncocytic features could even work synergistically with alterations in different genes (such as the PTEN/FLCN axis), adding an extra layer of complexity to inherited studies. The incomplete penetrance of the disease, the diverse phenotypes in the affected familial members (goiter, adenoma, thyroid nodules and carcinoma) and the association with syndromes are also confounding factors worth studying in the future. Much is yet to know, and we are still far from successfully establishing a pattern that could explain the genetic causative events in a majority of families/patients affected by a genetic predisposition to such tumors.

## Mutational Status of Mitochondrial Genome

The most common molecular feature of oncocytic tumors, in addition to the extremely high number of abnormal mitochondria (29, 30), is the prevalence of mutations in mtDNA genes, with a major incidence in genes encoding proteins of the mitochondrial respiratory complex I (12, 31–36). The high frequency of mtDNA mutations has advanced the so-called “compensatory theory” (37). Many of the mtDNA mutations found in oncocytic thyroid tumors lead to 1) a reduction (or even a total loss) of the expression of mitochondrial proteins (frameshift or non-sense mutations), or to 2) the alteration of aminoacids in those proteins (missense mutations) that can lead to protein loss of function. Ultimately, as a consequence of any of the aforementioned changes, there is loss of function of the corresponding mitochondrial respiratory complex (12, 37, 38). The “compensatory theory” proposes that the accumulation of mitochondria in oncocytic tumors emerges as a sort of an attempt of tumoral cells to compensate the loss of mitochondrial function leading to oncocytic change.

It has also been recently reported that autophagy mechanisms that are responsible for the elimination of impaired cellular components, including mitophagy – a particular autophagic mechanism for mitochondrial degradation – are altered in oncocytic tumors (31, 39, 40). The inefficiency of mitochondrial quality control mechanisms may also contribute for the accumulation of abnormal and dysfunctional mitochondria until the full blown eosinophilic cytoplasm is reached.

It is usually accepted that the damage and the occurrence of mutations in mtDNA may result mostly from the generation of reactive oxygen species (ROS) triggered from external insults (41, 42). Mitochondria are central organelles where ROS are produced and, thereby, is the first cellular location where damage occurs. In the nucleus, there is an intricate and complex machinery protecting and repairing DNA from damage. In the last decade, some DNA repair mechanisms were described in mitochondria similar to the ones existing in the nucleus (43), but it is still under investigation to which extent the reparation of mtDNA is effective. Besides the fact that mtDNA is more prone to ROS-induced damage, there are several copies of mtDNA *per* mitochondrion and there are several mitochondria within one cells’ cytoplasm, increasing the difficulty for disclosing the relevance and extension of the DNA repair mechanisms.

Another striking alteration reported in mtDNA in oncocytic tumors is the “common deletion”. This deletion encompasses a region of 4977bp, corresponding to almost one third of all mitochondrial genome. In a series of 16 oncocytic lesions, our group reported that all tumors harbored this deletion in 4-8% of all mtDNA molecules (44).

Despite the acknowledgement of the aforementioned molecular changes in oncocytic tumors, doubts remain regarding their clinical relevance, and have not yet been applied into clinical routine for diagnosis or prognosis. One of the limitations for their applicability is the lack of a mutational hot spot. The detection of one or more mutations in a range of 16.5 Kb, the length of the mitochondrial genome, limits the routine applicability for technical and cost reasons. In the context of both sporadic and familial tumors it is necessary to further study which mutations and mtDNA genes are associated with each entity in order to identify which ones may correlate with a worse prognosis. It would be good to allow the identification of small regions of the mitochondrial genome with a more favorable cost-effectiveness balance.

## Chromosomal Loss and Gains in Oncocytic Tumors

One of the most recently studied change in oncocytic tumors is the loss of heterozygosity, to a point of almost haploidy. Thorough analyses of somatic copy number alterations (SCNA) reported widespread chromosomal losses that are a hallmark feature of oncocytic tumors (45–49). Such alterations were associated with a poorer prognosis, suggesting that widespread chromosomal losses contribute to a more aggressive disease (45–49). SCNA analysis in HCC demonstrated that chromosomal haploidisation is frequent across almost all genome, with retaining heterozygosity in chromosomes 7, 5, 12 (45, 46) and 20 (46). In line with this, our group compared oncocytic and non-oncocytic PTC and reported higher chromosomal gains in the oncocytic group, including a large part of chromosome 7, and, in a less extent, chromosomal losses that occur mainly on chromosome 22 (31). Another study suggested that loss of chromosome 22 has guarded prognostic value for patient survival (50). One common determinant of the aforementioned studies is the fact that chromosome 7 evades LOH, suggesting that may be important for tumor progression (31, 45–48, 50).

SCNA studies suggest that there is a disruption of the machinery responsible for controlling the DNA damage repair in oncocytic tumors. Corver et al. advanced a possible explanation for such loss of heterozygosity in oncocytic tumors (47). *In vitro* studies using the oncocytic derived thyroid cell line XTC.UC1, the authors uncovered that decreasing ROS levels led to CHK2 downregulation and a consequent reduction in chromosomal segregation errors, while the opposite was also true, i.e. increasing levels of ROS led to an upregulation of CHK2 (47).

Alterations have been reported regarding proliferation and cell cycle in oncocytic tumors (51). In 2000, Erickson et al. reported that the expression of Ki-67 and cyclin D1 allowed to distinguish, to some degree, oncocytic adenomas from carcinomas (51). On the other hand, oncocytic tumors have a lower proliferative potential, another reason that contributes to the accumulation of mitochondria (30, 52). The implication of oxidative stress in oncocytic tumors goes beyond the impact on cell cycle regulation per se. ROS act as deleterious molecules that induce cellular damage in several (intra)cellular components and can act as secondary messengers. It was recently demonstrated that metabolic readjustments, and (in)balances of ROS intracellular levels, are related with genomic instability in oncocytic tumors (53). The aforementioned observations are relevant for the development of the oncocytic phenotype that were studied in the somatic context. It remains unknown whether alterations in oxidative stress and in chromosomal copy numbers may also be determinant for inherited oncocytic tumors.

The biogenesis of mitochondria in a high turnover context, as suggested above regarding GRIM-19 mutations, requires the upregulation of several proteins. Several proteins related to mitochondrial processes, such as mitophagy and mitochondrial dynamics, may also play a role in oncocytic changes. It has been reported an overall upregulation of mitochondrial dynamics proteins, in particular the upregulation of the mitochondria fission dynamin-related protein-1 (DRP1) in malignant oncocytic tumors (54, 55). Interestingly, the expression of the active form of DRP1, phospho-616-DRP1, was recently associated with locally invasive characteristics of TC, including lymph node metastases (56).

Acknowledging the existence of a number of major alterations (mitochondrial protein alterations, metabolic readjustments, compensatory mechanisms, oxidative stress, chromosomal instability and resistance to cell death), it still remains to understand the full picture of causes and consequences regarding the oncocytic change (52, 57–59).

## Primary or Secondary Oxyphilia

**Figure 1:**
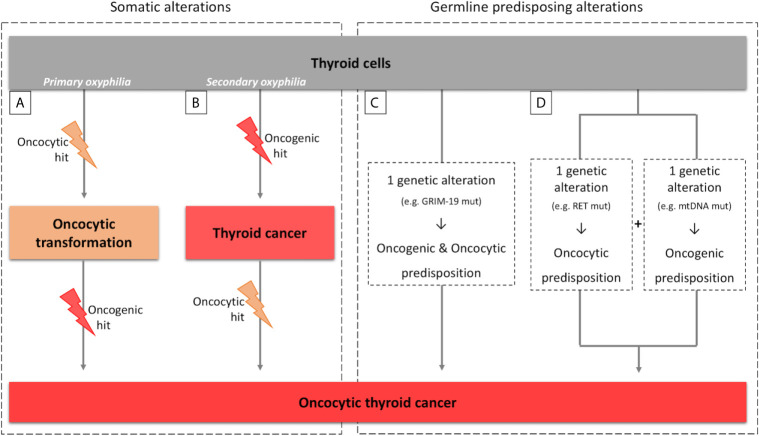
Parallelism between sporadic and hereditary tumors for primary/secondary oxyphilia. **(A, B)** scheme representing the primary and secondary oxyphilia concepts and the respective time of occurrence of the somatic oncogenic and oncocytic events; **(C, D)** scheme representing the primary and secondary oxyphilia in relation to the genetic predisposing alterations to oncocytic thyroid tumors.

The key question concerning the concept of primary versus secondary oxyphilia is related with the timing of oncogenic hit and additional oncocytic events in the establishment of the tumor (**Figure 1**) (60).

Oncocytic features of tumors can emerge at the time of, or previously to, the oncogenic hit (primary oxyphilia; **Figure 1A**), or can emerge as (a) secondary event(s), after the oncogenic hit (secondary oxyphilia; **Figure 1B**) (52, 61).

There are patients that present one (or more) oncocytic tumor(s). These findings fit with primary oxyphilia, assuming that the tumor(s) has (have) originated from neoplastic-initiating cells with a genetic predisposition for the oncocytic phenotype.

There are other patients with conventional tumors with some neoplastic clusters presenting oncocytic features, as well as patients with several concomitant tumors, some with and other without oncocytic features. These cases fit into secondary oxyphilia type of tumors, but some critical thinking may be necessary in order to clarify the mechanisms involved in oncocytic tumorigenesis.

While in medical terms the primary and secondary oxyphilia present as an apparent dichotomy, without translational significance, the biological mechanisms behind them could enlighten why in FNMTC context one sees an enrichment of oncocytic lesions.

A parallelism can also be made between the concept of primary and secondary oxyphilia and the events that predispose to thyroid carcinogenesis and oxyphilia in the hereditary context (**Figures 1C, D**). There are also at least two possible scenarios:

Scenario a) the oncocytic tumors occur due to one genetic alteration in a gene that simultaneously predisposes for emergence of tumor(s) and the oncocytic phenotype (**Figure 1C**). The TCO locus and the genes that have been associated with oncocytic tumors are examples of the aforementioned possibility. In particular, GRIM-19 is not only related to mitochondrial function but it is also related to the tumorigenic process itself (16, 19, 62);

Scenario b) two simultaneous genetic alterations may coincide in the same individual, one predisposing to tumorigenesis and another predisposing to the oncocytic phenotype (**Figure 1D**). An example was described by Abu-Amero et al. who demonstrated the presence of mtDNA mutations, most of them in genes that encode proteins from mitochondrial respiratory complex I, as well as RET germline mutations, in individuals with oncocytic familial medullary thyroid carcinoma (FMTC) or multiple endocrine neoplasia (MEN) type 2 (MEN2A) (63).

Whenever there are genetic alterations in mtDNA, even if they are secondary to a previous nuclear oncogenic event, one has to consider the possibility of other pathogenic factors contributing to the oncocytic change and the timing of its occurrence. Are the aforementioned alterations homoplasmic or heteroplasmic? In case of heteroplasmy (i.e., a mixture of wild type and mutated mtDNA molecules), what will be the levels of mtDNA mutation and penetrance for the disease to become phenotypically evident? Different factors may explain why there are oncocytic and non-oncocytic tumors in individuals belonging to families of FNMTC. There are a number of open questions whose answers will hopefully allow a better understanding of the etiopathogenesis of oncocytic tumors.

## Clinical Implications of the Oncobiology of Oncocytic FNMTC

Three clinical implications from what is known about the genomic, metabolism and proteomics related to oncocytic benign and malignant tumors, should be further studied within the familial context.

First, it is generally accepted that FNMTC evolves to a more aggressive phenotype than its sporadic counterparts, with a trend for younger age of onset, extrathyroidal invasion and lymph node metastasis (64). It raises the role of screening and early diagnosis that should be pursued whenever a family history is present, particularly in a syndromic context. Epidemiological data show a very high relative risk for first-degree relatives of index cases presenting with thyroid cancers (65–67). Additionally, benign thyroid disease is often observed in relatives of FNMTC individuals, and benign and malignant thyroid tumors in the same family displaying some extent of cell oxyphilia, pointing to the need to investigate any relatives with sporadic NMTC with oncocytic features (14). We suggest that it is necessary to assess family members from patients with FNMTC for thyroid lesions in routine examination, since first degree of probands with thyroid cancer may be underestimated in current guidelines (65). The screening of kindred for the appearance of new thyroid lesions, taking advantage of ultrasound and the minimally invasive fine-needle aspiration (FNA), is not yet recommended by guidelines due to insufficient evidence supporting the reduction of morbidity and/or mortality (68). Screening measures are warranted for syndromic cases as the ones previously described, based on various elements of a particular syndrome in any first-degree relative (69). Noteworthy, an enrichment of oncocytic lesions has recently been described as associated with the DICER 1 germline mutations (70). It may be reasonable to start considering investigating DICER 1 variants in index cases of oncocytic FNMTC, as well as in direct relatives for whom such oncocytic lesions have been detected in screening procedures, as part of the genetic counseling in a syndromic context.

Second, there is a high unmet need for biomarkers on cytology samples from FNA capable of identifying oncocytic thyroid lesions and, furthermore, to evaluate the malignancy risk in such cases. Finding oncocytic tumor markers would be most welcome, namely its clinical application in the FNMTC context, a setting that seems quite unexplored.

Third, HCC seems to behave biologically different from follicular carcinoma. HCC tends to metastasize more to lymph nodes, has a lower avidity to radioiodine, and a higher rate of recurrence and tumor-related mortality (68, 71–74). Incidentally, it has been demonstrated that diagnosis of lymph nodes metastases, in the setting of HCC, represent frequently soft tissue metastases arising from venous invasion (75). Lymph node metastases (LNM) at the time of diagnosis are more frequent in HCC as compared with FTC (76–78). When evaluating the presence of LNM from oncocytic neoplasms, it is also important to acknowledge that some oncocytic tumors are indeed variants of PTC, and not variants of follicular carcinoma (so called HCC), therefore justifying the discrepancy of LNM rates among different reports about oncocytic tumors. The lower avidity to radioiodine is a characteristic of HCC tumors widely recognized by those who treat them, and has been documented in various studies (68, 79, 80). In particular, less than 40% of patients who present with or evolve to lymph node and/or distant metastases respond to radioiodine (80). Recurrence rates can vary from 14% up to 44% (76, 78, 81–84), and this wide range speaks for the retrospective nature of all published studies and potential different histological classification criteria, as was highlighted in a recent review by our group (85). Nevertheless, if HCC patients present with distant metastases, the mortality rate can reach 80% (86).It is generally acknowledged that oncocytic tumors are typically slowly proliferative, more resistant to apoptosis, and more tendency to necrosis, as if they are not able to activate the ultimate cell fate – programed cell death (61). Despite this, there are no specific recommendations for the treatment of HCC and its management is similar to that of FTC, in the same way oncocytic variants of PTC are treated as conventional PTC with identical stage in standard guidelines (68, 72).

Biomarker-based algorithms that could predict pre and postsurgical TC prognosis and guide surgical approaches, radioiodine adjuvant treatment need and dosage, TSH suppression level, as well as new therapy approaches to revert radioiodine refractoriness are needed in HCC, a fact that goes beyond the hereditary setting. Currently, no recommendation for molecular analysis exists to support the diagnosis of oncocytic tumors. Further research and disclosure of the molecular pathways that could confer tumoral advantage to oncocytic tumors is required to understand if molecular analysis could translate into practice treatment and follow-up decisions. Scarce information regarding the signaling cascades of apoptosis/necrosis in oncocytic tumors is available. Elucidation on how, and in which degree, cell death related mechanisms are altered in oncocytic tumors would potentially offer a therapeutic opportunity. Another missing molecular piece concerns the radioiodine refractoriness of oncocytic tumors. Disclosure of altered molecular pathways that contribute to treatment resistance may offer new targetable therapeutic options to improve the efficacy of this standard of care therapy in TC. Some studies have been demonstrating that MAPK and TGF-β1/Smad/FoxP3 are involved in the re-differentiation of thyroid tumors and in the re-expression of the sodium-iodine symporter (NIS, also known as solute carrier family 5 member 5 - SLC5A5) in non-oncocytic tumors (87–89). Understanding the contribution of these signaling pathways in NIS expression and membrane localization in oncocytic tumors would be relevant in the identification of the patients who will most likely not respond to radioiodine, and thereby require different treatment strategies. Most importantly, there is a need for molecular biomarker(s) that could predict which forms of HCC (e.g., encapsulated HCC) will metastasize. Discrimination between minimally invasive and widely invasive HCC has prognostic implications, but the fact that it can only be done after surgery results in a lost opportunity to optimize clinical choices early in the treatment algorithm. Some recent efforts have been done in this field, but much is still unknown (45, 90).

## Conclusions

The understanding of the mechanisms behind the inheritance of TC with oncocytic features are still unclarified, similarly to what happens with the broader FNMTC group displaying incomplete penetrance and variable phenotypes. Genetic events increasing susceptibility to oncocytic phenotype have been mapped to the TCO locus demonstrating the association between a germline mutation and the oncocytic phenotype. While other candidate molecular (genetic) mechanisms are being explored within and outside the familial context (**Table 2**), the mechanistic association between genomic changes, metabolic alterations and mitochondrial protein expression will contribute to establish driving forces that trigger and sustain the oncocytic change. Hopefully, these advances will allow to progress along the above mechanisms and to provide a translational application, not only in early diagnosis, but also in terms of treatment.

**Table 2:** Summary of the major alterations reported in oncocytic thyroid tumors.

	nDNA (nuclear DNA)		mtDNA (mitochondrial DNA)			SCNA (somatic copy-number alterations)	
	Hereditary associations (gene mutation) (14, 16–18)	Somatic gene mutations (45, 46)	Mutations/deletions in mitochondrial DNA genes (31, 33, 36, 91–94)			Overall chromosomal losses (31, 46, 48, 49, 95)	
	TCO locus GRIM-19 TIMM44 MYO1F (**Table 1**)	DAXX, EIF1AX, MADCAM1, NF1, p53, TERTp, others (<10%)	Mutations with particular enrichment in the complex I	Mutations in the complexes III/IV and ATPase	Common deletion	Wide-ranging loss of heterozygosity (LOH)	Retention of diplopy (or duplication) in chromosomes 5, 7, 12 and 20
***Consequences***	Predisposition to thyroid cancer with cell oxyphilia	Thyroid cancer formation	Loss of assembly and function of mitochondrial respiratory complexes;			Genome haploidisation	
		? Early TC event?	OXPHOS impairment;			Tumor suppressor genes inactivation (gene mutation + LOH)	
			??? Impairment of other mitochondrial functions (apoptosis, oxidative stress,…)				

## Author Contributions

All authors contributed to conception of the manuscript. MC, AL, and RB made literature research and wrote the first draft of the manuscript. VM and MS-S made the first manuscript revision and edited the manuscript. All authors contributed to the article and approved the submitted version.

## Funding

This work was supported by SPEDM (Portuguese Society of Endocrinology, Diabetes and Metabolism; grant code MITODI: Diabetes mellitus and oncocytic tumors of thyroid: the mitochondrial connection.”) and by EISAI (grant code ROMITO-DRP1: “ROle of the MITOchondrial fission protein Drp1 as a prognosis and predictive biomarker in the treatment of differentiated thyroid cancer”).

## Conflict of Interest

The authors declare that the research was conducted in the absence of any commercial or financial relationships that could be construed as a potential conflict of interest.
